# Load Auditory Feedback Boosts Crutch Usage in Subjects With Central Nervous System Lesions: A Pilot Study

**DOI:** 10.3389/fneur.2021.700472

**Published:** 2021-07-06

**Authors:** Federica Tamburella, Matteo Lorusso, Nevio Luigi Tagliamonte, Francesca Bentivoglio, Alessandra Bigioni, Iolanda Pisotta, Matteo Lancini, Simone Pasinetti, Marco Ghidelli, Marcella Masciullo, Vincenzo Maria Saraceni, Marco Molinari

**Affiliations:** ^1^Spinal Rehabilitation Laboratory (SPIRE Lab), Neurorehabilitation 1 Department, Santa Lucia Foundation, Rome, Italy; ^2^Laboratory of Robotic Neurorehabilitation (NEUROROBOT Lab), Neurorehabilitation 1 Department, Santa Lucia Foundation, Rome, Italy; ^3^Advanced Robotics and Human-Centered Technologies Research Unit, Università Campus Bio-Medico di Roma, Rome, Italy; ^4^Deptartment of Mechanical and Industrial Engineering, University of Brescia, Brescia, Italy; ^5^Rehabilitation Center, Turati Foundation, Zagarolo, Italy

**Keywords:** auditory feedback, crutches, gait, rehabilitation, adherence

## Abstract

**Background:** Crutches are the most common walking aids prescribed to improve mobility in subjects with central nervous system (CNS) lesions. To increase adherence to the appropriate level of crutch usage, providing load-related auditory feedback (aFB) may be a useful approach. We sensorized forearm crutches and developed a custom software to provide aFB information to both user and physical therapist (PhT).

**Aim:** Evaluate aFB effects on load control during gait by a self-controlled case series trial.

**Methods:** A single experimental session was conducted enrolling 12 CNS lesioned participants. Load on crutch was recorded during 10 Meter Walk Test performed with and without aFB. In both cases, crutch load data, and gait speed were recorded. Usability and satisfaction questionnaires were administered to participants and PhTs involved.

**Results:** Reliable data were obtained from eight participants. Results showed that compared to the no FB condition, aFB yielded a significant reduction in the mean load on the crutches during gait (*p* = 0.001). The FB did not influence gait speed or fatigue (*p* > 0.05). The experience questionnaire data indicated a positive experience regarding the use of aFB from both participants' and PhTs' perspectives.

**Conclusion:** aFB significantly improves compliance with crutch use and does not affect gait speed or fatigue by improving the load placed on crutches. The FB is perceived by users as helpful, safe, and easy to learn, and does not interfere with attention or concentration while walking. Furthermore, the PhTs consider the system to be useful, easy to learn and reliable.

## Introduction

Walking is a fundamental human activity (1); when it is affected by illness or injury, people prioritize regaining the ability to walk as a goal of treatment (2). Up to 10% of adults suffer from reduced mobility or balance as a result of conditions, such as a central nervous system (CNS) lesions, which affect balance and gait. In Europe, walking aids are the most commonly prescribed tools to improve balance and mobility in this population (3, 4). Notably, the number of individuals using crutches and assistive devices for mobility is growing rapidly (4).

The main uses of crutches are to better support one's weight (by reducing the magnitude of load on the legs), and to improve balance (by increasing the body's base of support) (5). Patients typically receive training on how to use walking aids during rehabilitation sessions. Clinicians guide the usage of crutches according to patients' functional and recovery states. For example, they ask the user to progressively decrease the load on crutches as they improve motor performance or become more acquainted with a new prosthesis/overground exoskeleton.

Assessing crutch use is critical to ensure the crutches are used properly and to avoid overuse of the upper limbs (4). Clinical assessments focus on the magnitude of support weight and balance control (1). Due to a lack of objective measurements, these assessments performed in daily clinical practice remain subjective and qualitative. The availability of objective, quantitative data on crutch use may improve rehabilitation treatments (5, 6).

Researchers have proposed the introduction of sensors in crutches to monitor several variables, such as upper limb joint forces and torque, the axial load on crutches and their orientation (1, 7, 8). For instance, in (7–11), instrumented crutches were used to objectively monitor the load on the lower limbs of healthy subjects (8, 10, 11) or patients in a rehabilitation framework (7, 9, 12). Other applications include monitoring crutch use during daily life (10) or domestic environments (9), estimating physical activity, performing clinical diagnoses, or monitoring gait training (13).

To improve the ability of individuals to walk with crutches, approaches based on feedback (FB) have been proposed to ensure proper crutch use in individuals with different orthopedic (14) and neurological clinical diseases. Regarding the latter group of individuals, specific tests have been conducted in multiple sclerosis (1) or spinal cord injury (7, 15) subjects. FB is, at present, considered the main approach to guide top-down control mechanisms and to drive recovery, particularly when dealing with external devices (16). FB and traditional physiotherapy complement each other in assisting the patient functional recovery (17). A range of FBs, adapted to user's individual needs and residual functional abilities (18), can be used with the aim to boost neuroplasticity in neurorehabilitation. The FB can be provided in real-time *during* the execution of a task (concurrent FB) or soon *after* (terminal FB). Mainly two types of concurrent FBs are available for clinicians: “biofeedback,” that refers to biological signals about which the subject is partially/completely unaware, and “augmented feedback,” i.e., a FB given by a device on measures about which the subject is already directly aware (19). In this scenario, different signals can be used to feed FB information, but at present no indication exists for their specific effects on performance (17, 19). Nonetheless, it has been demonstrated that new technologies based on different FB modalities (19), such as visual (20–22), acoustic (23) and/or haptic (24, 25), allow re-education of altered functions (26, 27), and a consensus is forming on the role of FB to guide and improve patient-technological device interactions (16).

The aspects of FB that are important for guiding and improving patient performance include motivation, active participation, and error-driven learning (28). Consequently, patients must be aware of the differences between real-time results and the desired expected performance (28). The possibility of exploiting FB to compare the actual outcomes with expected outcomes in real time may positively affect motivation and self-efficacy, and may motivate participants during training (29).

In this pilot study, we sensorized forearm crutches, one of the most commonly used types of crutches (4). Strain gauges were added at the base of the crutches to monitor crutch-ground axial interaction forces. Moreover, custom software was developed to provide a concurrent auditory FB when prescribed load limits were exceeded during walking.

In this pilot study, we analyzed the capability of CNS-lesioned participants to adhere to a performance target defined for gait rehabilitation by using traditional crutches and sensorized crutches with auditory FB. In particular, the target for the participants was a reduction of the load applied on the crutches during gait. We compared participants' performance with and without the auditory FB information, and we assessed adherence to the walking target in terms of the load placed on crutches, gait speed and related fatigue. The main goal was to determine whether auditory FB information can improve participants' adherence to the imposed target. We also evaluated the participants' experience with the auditory FB and PhTs' perception of the usability of, and satisfaction with the sensorized crutches and the software.

## Materials and Methods

This pilot study was a self-controlled case series trial. The protocol was written according to the Helsinki declaration and approved by the Independent FSL Ethics Committee (Prot. CE/PROG.741). Written informed consent was obtained from all participants according to the FSL ethical procedures.

### Enrolled Participants

A convenience sample of 12 participants admitted to the Neurorehabilitation 1 Department of Fondazione Santa Lucia (Rome, Italy) and to Fondazione Turati (Zagarolo, Italy) from May 2019 until December 2019 was recruited. Data from four participants were excluded due to the presence of noise in the sensors; consequently, the final sample size was equal to eight. The inclusion criteria were (i) subacute or chronic stroke, spinal cord injury (SCI) and multiple sclerosis (MS); (ii) the ability to walk with one or two crutches for at least 10 m; (iii) a FAC (30) score ranging between 3 and 5 for the stroke and MS participants; (iv) a WISCI (31) level ranging between 9 and 19 for the SCI participants; and (v) the ability to understand verbal instructions. The exclusion criteria were as follows: (i) cognitive or behavioral impairments interfering with the comprehension of instructions; (ii) severe disturbances of the auditory system; (iii) severe concomitant diseases; and (iv) the inability to provide informed consent. The participants' epidemiological, clinical and neurological features are reported in Table 1.

**Table 1:** Epidemiological, clinical, neurological data, and experimental conditions for each participants (P1–P8).

	**P1**	**P2**	**P3**	**P4**	**P5**	**P6**	**P7**	**P8**
**Epidemiological, clinical and neurological data**								
Age	23	52	21	45	64	51	39	45
Gender	M	M	M	F	M	M	F	M
Weight [kg]	51	81	63	60	84	75	70	80
CNS lesion	Traumatic SCI!!break (C5 AIS D)	Traumatic SCI (C4 SCI AIS)	Traumatic SCI (C5 AIS D)	SM	SM	Stroke	Stroke	Traumatic SCI (L4 AIS D)
Days since lesion	119	113	143	5458	1806	104	92	365
**Experimental condition**								
Routine aids	Walker	Walker/AFO	Human assistance	2 crutches	2 crtuches/AFO	Walker	Walker	2 crtuches/AFO
Number of crutches	2	1	1	2	2	1	1	2
Crutch pattern	4	2	2	2	4	2	2	4
Crutch (es) with FB	1	1	1	2	1	1	1	1

### Walking Target

All participants were undergoing rehabilitation training at the time of the experiment, with the main goal of improving gait abilities by reducing the load over the aids as much as possible in a safe way. Therefore, the walking rehabilitation objective during the experiment was a reduction in the load applied on the crutches during a walking task at a self-selected comfortable speed.

#### Auditory Feedback Approach

To provide participants with the auditory FB, instrumented crutches with a peak detection algorithm were employed, as detailed in section Wireless Instrumented Crutches. The general approach to monitor the load and generate sounds accordingly was as follows:

1. Three threshold values were defined from the data collected during a baseline evaluation: the lowest threshold *Th*_min_ only discriminated the stance and swing phases, while the other two thresholds, *Th* and *Th*_*MAX*_, indicated excessive load values. For this reason, the three thresholds were set as the 40th, 82nd, and 97th percentiles of the load distribution.
2. Different sounds were triggered when these thresholds were exceeded: the crutches produced no sound when the load was below *Th*_min_ (stance phase) and between *Th*_min_ and *Th* (participant applies correct loads), a low-pitched tone when the load was between *Th* and *Th*_*MAX*_ and a high-pitched tone when the load exceeded *Th*_*MAX*_.

### Experimental Design

#### Wireless Instrumented Crutches

**Figure 1:**
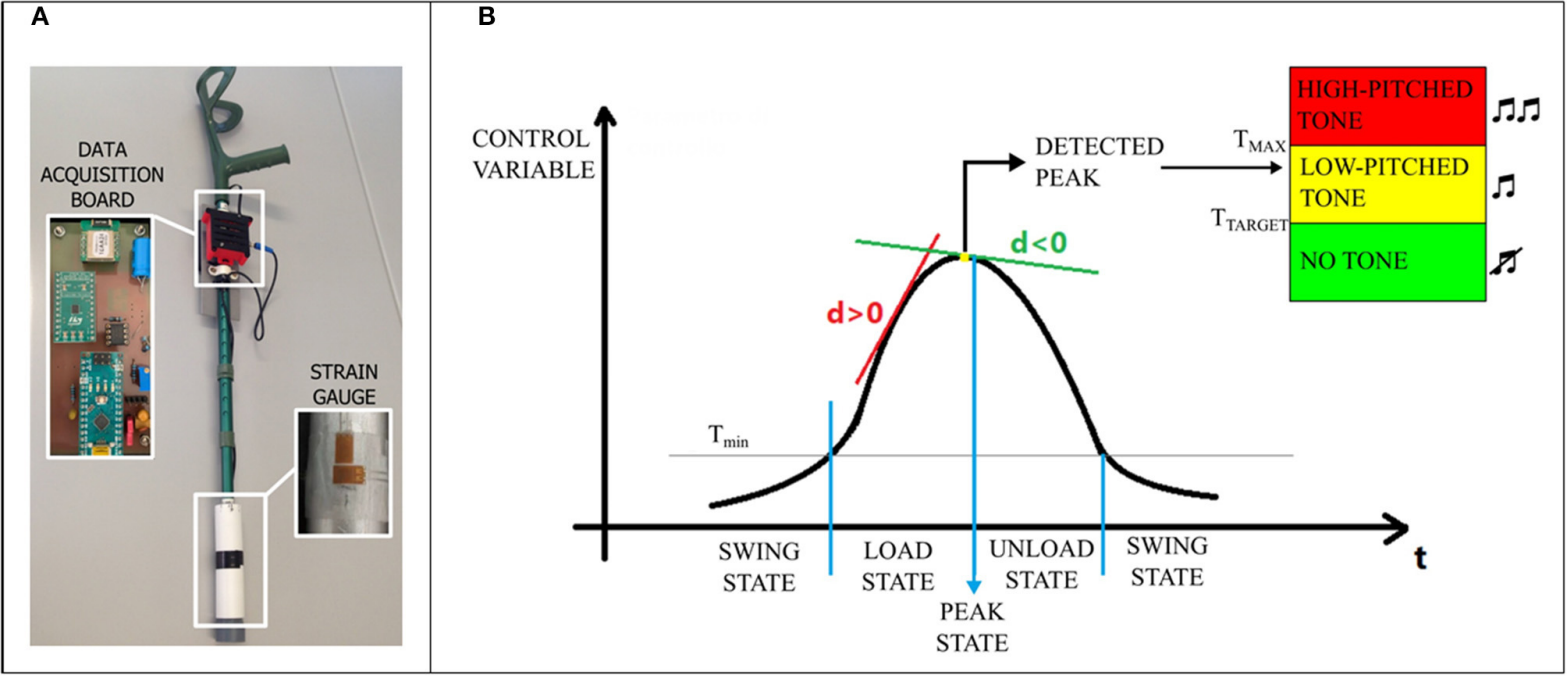
Instrumented crutch **(A)** and peak detection algorithm description **(B)**.

A pair of instrumented crutches capable of measuring the real-time axial forces, with a sensitivity of 0.005 V/N in the range of 0–600 N (7, 10) was used in all the trials (simultaneously or individually). Each instrumented crutch (Figure 1A) was equipped with one strain gauge full bridge (nominal resistance: 120Ω); a data acquisition board composed of a microcontroller (Arduino Nano) for data acquisition, an AD converter (10 bit resolution) for strain gauge bridge conditioning, an inertial unit (LSM9DS1) for detecting the impact with the ground, and a Bluetooth module (ESD200) for wireless data transmission; and a battery power supply. The electronic board was attached to each crutch by using a removable box and connected to the strain gauges through a detachable flat cable. Force data were acquired at 50 Hz and sent in real time to a client PC by using a custom virtual instrument (VI) developed in LabVIEW™ (National Instruments). The LabView VI was used for data processing and visualization.

#### Experimental Session

Data were collected during a single experimental session. Each session included the following phases:

- During the baseline evaluation, the participants were asked to comfortably walk at their self-selected velocity with one or two crutches, according to their training protocol, along a 10 m path for the 10 Meter Walk Test (10MWT) (32); the test performance and the load on the crutches were recorded. For the 10MWT a clear straight pathway of 20 m in length over solid flooring was required. Clear marks at the start and end point of 20 m walkway were applied, as well as two more marks at 5 and 15 m to identify the central 10 m path to be timed by PhT with a stopwatch. Excluding from measurement acceleration and deceleration, that occur outside of the timed portion, allows to only assess steady-state walking speed. During test execution, the patient was instructed to start on the 0 m mark, walk at her/his own comfortable walking speed and stop when reaching the farthest mark (32). Based on the load data, the load thresholds *Th* and *Th*_*MAX*_ were set to be used during the subsequent testing phases. No verbal instructions from PhTs were provided.
- For the familiarization phase (10 min), the participants were asked to walk both with auditory FB and PhT verbal instructions and with PhT verbal instructions only without FB. These instructions aimed to promote a more physiological gait pattern with a little load as possible on the crutch (es). During familiarization, as well as in the testing phase, the participants received different auditory FB related to the load, as detailed in section Auditory Feedback Approach. The participants rested for 5 min before continuing to the next phase.
- During the testing phase, the participants walked with the sensorized crutches three times with FB and three times without FB (noFB condition) in a randomized order, and 10MWT performance was recorded. A PhT was present for safety reasons, but no verbal instructions were provided in either condition, with or without FB. At the end of each of the six runs, the BORG scale (33) was administered to the participants.
- During the experience assessment, user-related data were acquired by a specific questionnaire developed on the basis of previously validated data (34, 35). The PhTs' perspectives about the system (FB software and crutches) were evaluated with the System Usability Scale (34) and the Quebec User Evaluation of Satisfaction with assistive technology 2.0 (35).

#### Data Extraction and Analysis

A peak detection algorithm was used to identify the maximum value of a signal selected by the experimenter (namely, control variable, CV) in real time during each gait cycle. The available CVs include the axial force on a single (right or left) crutch or the mean value calculated between the left and right axial forces. The peak detection algorithm identifies the peak of each gait cycle *F*_*peak*_ by computing and analyzing the first derivative *d* of the CV. A state machine approach was used as described in Figure 1B. Four different states were defined: (i) the SWING state, (ii) the LOAD state, (iii) the PEAK state, and (iv) the UNLOAD state. The SWING state was activated when the CV decreased below the minimum threshold *Th*_min_ (experimentally set as the 40th percentile of the distribution of the CV). When the SWING state was activated a new stride was segmented. When the CV exceeded *Th*_min_, the LOAD state was activated (*d*>0). The PEAK state was activated when *d* changed its sign, and the peak was identified as the current value of the CV. Then, the UNLOAD state was activated (*d* < 0). When the signal decreased below *Th*_min_, the peak search was reset, and a new peak search started.

When the PEAK state was activated, the algorithm compared the CV with two other thresholds, *Th* and *Th*_*MAX*_ (experimentally set as the 82nd and 97th percentile of the distribution of the CV, respectively), and generated a single-tone sound whose frequency *f* was set based on the following rules:

- *f*= 0 Hz (no tone) if *F*_*peak*_ < *Th*;
- *f* = 440 Hz (low-pitched tone) if *Th* ≤ *F*_*peak*_ < *Th*_*MAX*_;
- *f* = 880 Hz (high-pitched tone) if *F*_*peak*_ ≥ *Th*_*MAX*_.

*Th* and *Th*_*MAX*_ were considered limits for the participants, who were asked to walk without generating sounds.

The following variables were extracted by averaging the values from three runs for each FB condition:

- For the load data, we calculated the following metrics: $\bar{F}$ - mean peak load on crutch (es), i.e. the average of the peak values identified in a walking path; ${\bar{F}}_{\%}$ - percentage of variation of $\bar{F}$ with respect to the target threshold (*Th*); *N*_*TOT*_ - number of crutch contacts on the ground (i.e., number of load peak values); and *N*_*Th*_ - percentage of peak values lower than *Th*.
- To determine gait speed, the 10MWT (m/s) was administered. The total time taken to walk in the central 10 m path was recorded and the speed was consequently calculated.
- To determine fatigue, the Borg scale for fatigue was administered.
- For the experience data, an *ad hoc* FB questionnaire composed of 9 items was administered. It was developed to assess 4 factors (Table 2): *utility/usability* (items 1, 4, 5, 6, 7), *safety* (item 3), *attention* (items 2, 9), and *learnability* (item 8). The participants used a 7-point Likert scale (36), ranging from 1 (“*I strongly disagree*”) to 7 (“*I strongly agree*”), to respond to each item. The wording of item 2 (*attention*) was reversed, which means that its meaning was opposite to the construct of interest (37).

**Table 2:** List of items included in the *ad-hoc* feedback questionnaire.

	***Ad-hoc* feedback questionnaire**
1	The auditory feedback was helpful during the training
2	The auditory feedback distracted me during the training (R)
3	The auditory feedback helped me to feel safe
4	The auditory feedback helped me to walk
5	The auditory feedback helped me to reach the goal planned by the PhT
6	Thanks to the auditory feedback, I was able to execute the instructions given by the physiotherapist more easily
7	If I had the opportunity to receive a feedback during daily life, I would be able to walk better
8	I think that the familiarization phase was enough to get me to handle the use of the crutches with the auditory feedback
9	When I walk with the crutches, I can focus on the necessary movements to walk

The System Usability Scale (SUS) was administered to the PhTs to evaluate *usability*, whereas the Quebec User Evaluation of Satisfaction with assistive technology 2.0 (QUEST 2.0) was used to assess *satisfaction* with the system. The SUS is a 10-item questionnaire rated on a Likert scale from 1 to 5, particularly used for the assessment of usability, perceived ease of use, and complexity. It was designed to evaluate a wide variety of products and services, including hardware, software, mobile devices, and websites (34). QUEST 2.0 is a 12-item standardized assessment tool with five response options. It is the most widely used questionnaire to evaluate satisfaction with assistive technologies. It provides direct data on user interactions with technology and is used to determine how useful and acceptable a technology is perceived in various settings. QUEST 2.0 has limited applicability with prototypes, so in our case, not all the items were used. The questionnaire consists of two parts: the first part is for the rating of the device's characteristics (dimensions, weight, adjustments, safety, durability, simplicity of use, comfort, and effectiveness), whereas the second part evaluates the services with five items. We only assessed the first part since no services were provided (38). After the rating procedure, a list with the satisfaction characteristics was presented to the user, and he or she was asked to choose the three most important ones (35).

#### Statistical Analysis

Statistical tests were performed by using SPSS software (Statistical Package for the Social Sciences–Chicago, IL, USA). Differences between the FB and noFB conditions were assessed with the paired *t*-test for the parametric variables (load data, 10MWT) and by the Wilcoxon test for the non-parametric variables (Borg data). Statistical significance was indicated when *p* < 0.05.

## Results

The data for six males and two females (age: 42.5 ± 14.6 years, mean time from lesion: 2.74 ± 4.99 years) were analyzed. The participants were assigned identifiers from P1 to P8. The aids used by the participants for routine gait are reported in Table 1, as well as the details of the experimental conditions, such as the FB source, number of crutches used and crutch gait pattern during testing. Indeed, there are multiple ways to walk with crutches, depending on the specific injury or disability. The points of contact or contact patterns indicate the basic structure of gait with crutches, as they reflect the number of times the crutches leave the ground and land within one gait cycle in the direction of walking (4).

### Load Data

**Figure 2:**
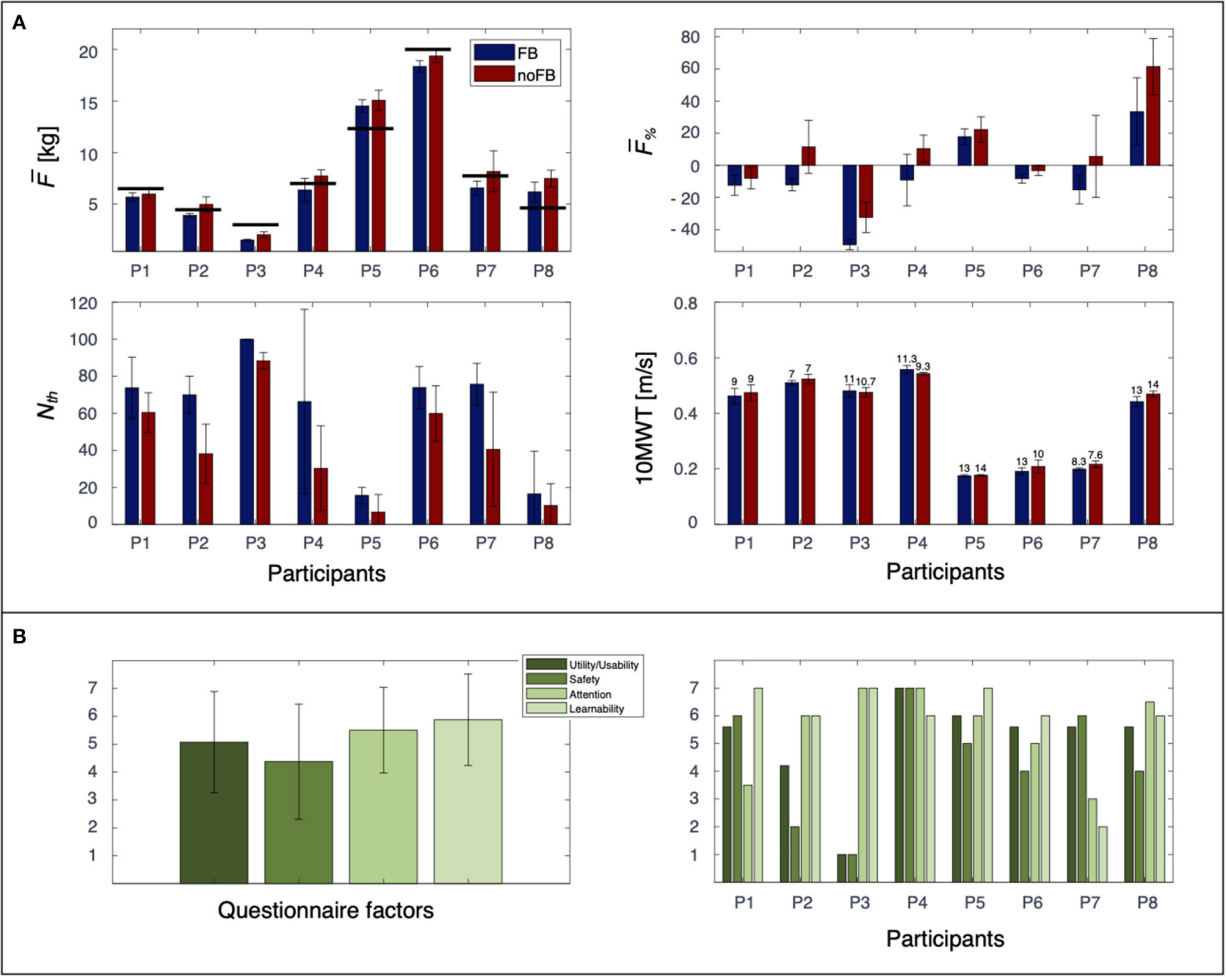
Load and 10MWT data for P1–P8 are reported in **(A)**. Error bars indicate standard deviation. The target threshold *Th* for each participant is represented by black horizontal lines in the $\bar{F}$ graph and by the zero value in the ${\bar{F}}_{\%}\;$graph. BORG values, averaged over three runs, are reported on top of the bars in the 10MWT graph. Overall experience with the auditory FB for each factor is reported in **(B)** as mean across participants (left) and separately for each participant (right).

For all participants, the mean load on the crutches $\bar{F}$ was significantly smaller by ~0.9 kg (*p* = 0.001) in the auditory FB condition than in the noFB condition (FB $\bar{F}$: 7.9 ± 6.03 kg; noFB $\bar{F}$: 8.8 ± 6.06 kg) (Figure 2A).

Interestingly, a different behavior was observed in terms of the FB effects related to ${\bar{F}}_{\%}$ for the participants. P1, P3, and P6 were able to load the crutches with loads smaller than the selected target for both the FB and noFB conditions, even when $\bar{F}$ was lower with FB. In contrast, P5 and P8 did not exhibit loads below the threshold for either the FB or noFB condition, although the load on the crutches was lower in the FB condition. For P2, P4, and P7, the presence of the FB reduced only ${\bar{F}}_{\%}$. Across the participants, ${\bar{F}}_{\%}$ was significantly smaller by ~15% (*p* = 0.002) with FB than without FB (FB ${\bar{F}}_{\%}$: −6.9 ± 19.64%; noFB ${\bar{F}}_{\%}$: 8.5 ± 17.75%) (Figure 2A). In detail, three participants (P1, P3, and P6) exhibited low ${\bar{F}}_{\%}$ values for both conditions, and two participants (P5 and P8) had low values that were not smaller than *Th*.

Auditory FB did not influence the participants in terms of *N*_*TOT*_ (*p* > 0.05). Indeed, no differences were present in the comparison FB vs. noFB (FB: 13.6± 3.66 peaks; noFB: 13.3 ± 3.22 peaks). Even though no differences in *N*_*TOT*_ between the FB and noFB conditions were observed, for all participants, *N*_*Th*_ (i.e., correct load) was higher with FB (Figure 2A). This finding was particularly evident for P2, P4, and P7, who were able to maintain ${\bar{F}}_{\%}$ only with FB. The percentage of peaks under the selected threshold *N*_*Th*_ with FB was 19.7% higher than that under the noFB condition (*p* = 0.003) (FB *N*_*Th*_: 61.5 ± 25.5%; noFB *N*_*Th*_: 41.8 ± 26.07).

### Gait Speed Data

No significant differences were observed in the comparison of the FB vs. noFB conditions for gait speed (*p* > 0.05 – FB 10MWT: 0.38 ± 0.18 m/s; noFB 10MWT: 0.39 ± 0.17 m/s) or the Borg score (*p* > 0.05 – FB Borg: 10.7 ± 2.2; noFB Borg: 10.2 ± 2.46) (Figure 2A).

### Experience Data

Overall, the participants responded positively regarding the use of the crutches with the auditory FB (Figure 2B). In fact, the mean score for all items was higher than 4, which is the intermediate score of the questionnaire. In detail, the participants perceived the auditory FB as useful (*utility/usability* score: 5.07 ± 1.81) and easy to *learn* (*learnability score:* 5.87 ± 1.64), with no impact on concentration while walking (*attention* score: 5.5 ± 1.53). *Safety* had a lower score (4.37 ± 2.06), suggesting that auditory FB might not have had a high impact on participants' perceived safety when they used the crutches.

Interestingly, although the general response was positive according to the questionnaire, different behaviors were observed from the individual participants (Figure 2B). In detail, the factor distribution showed large variability in the way participants perceived auditory FB. P4 and P5 gave high scores for all the factors in line with good load reduction in the FB condition. P3 gave very high scores for *attention* and *learnability*, while the *utility/usability*, and *safety* scores were low. As shown in Figure 2A, P3 had a value of 100% of peaks under the selected threshold: he never heard the auditory FB during the experimental session, which may have prevented him from being able to properly evaluate its utility and safety. P2 and P7 presented an opposite trend in questionnaire answers (P2 gave high scores for *attention* and *learnability* and low scores for *utility/usability* and *safety; P7* gave high scores for *utility/usability* and *safety* and low scores for *attention* and *learnability*), although they both showed the same behavior during gait (reduced the load the on crutches under the selected threshold only in the FB condition).

Regarding the PhT point of view, the SUS score was 87.5 (± 13.2) out of 100, indicating that PhTs perceived the system as useful, easy-to-learn and reliable. Furthermore, the QUEST 2.0 results indicated that PhTs were satisfied with the use of the system (4.38 ± 0.37 out of 5), and the three most important *satisfaction* items were weight, ease-of-use and effectiveness.

## Discussion

The significant load reduction in the FB testing condition demonstrates the usefulness of the peak load auditory FB during walking with crutches. Forearm crutch-assisted gait is frequently used in clinical settings for rehabilitation in individuals with CNS lesions (28, 39, 40). The amount of body weight that should be loaded on the crutch (es) depends on the pathology and the recovery phase that the participant is in (41, 42). At the time of the experiment, all participants were receiving rehabilitation with the goal of reducing the load on the aids as much as possible to improve their ability to walk independently. The crutches developed for this research are a type of wearable rehabilitation technology, and these technologies have been receiving increasing interest and offer advantages over traditional rehabilitation services (43, 44), such as lower costs, a wider range of applications, remote monitoring and greater comfort (29).

In this pilot study, we performed a single experimental session using a concurrent auditory FB and did not assess crutch usage longitudinally, and we asked participants to walk with instrumented aids at their self-selected comfortable speed. According to Agresta et al. (45), concurrent (auditory or visual) FB is one of the most effective, and it has been demonstrated that it produces the best short-term results (18). In line with these evidences, even without dedicated training for the use of the FB, all participants reduced the mean load on the crutches more when auditory FB was present. Furthermore, even though the number of times the crutches contacted the ground did not vary between conditions, the mean value of the number of correct peaks (i.e., peaks with a value below the selected threshold) was significantly higher when the participants received auditory FB. Moreover, 10MWT gait speed and fatigue perception did not vary between trials. These results suggest that the presence of the auditory FB did not affect gait speed or the number of times the crutches contacted the ground. Overall, these results appear particularly encouraging since, even in the absence of dedicated training, a single-use session still allows an immediate significant variation in the mean load on the crutches during gait. It is then expected that specific prolonged training could potentially further enhance load control.

This finding is confirmed by the subjective responses of the participants, which despite heterogeneity, were generally positive. One of our main concerns was that the FB could overload participants' attention by interfering with their ability to handle the load on the crutches. Instead, auditory FB did not interfere with the participants' attention while walking, and it was perceived as useful. Additionally, the PhTs showed a high level of satisfaction and a positive attitude toward the system, as it was perceived as useful and easy to use. These data indicated positive responses regarding the use of the auditory FB with the sensorized crutch system in the rehabilitation environment from both the participants and PhTs. Furthermore, these data suggest that PhTs trust the system and that it meets their planned rehabilitation objectives for CNS patients, such as improving the support of weight and balance. This is in line with the clinical professionals' needs for rehabilitation systems, which should be easy to use (18, 46) and applicable in the everyday clinical practice to improve the functional recovery process (14, 24).

In this work, we analyzed CNS-lesioned participants' adherence to crutch use during gait. Good adherence implies that the patients and physicians collaborated well to improve patient health (47). It has been demonstrated in CNS lesions that PhT-participant interactions are important for the success of rehabilitation. Physical, verbal, and technical exchanges between the PhT and participant highly influence the outcome, suggesting the importance of collaborative work in the rehabilitation framework (48). Moreover, increasing the effectiveness of adherence interventions may have a far greater impact on the health of the population than any improvement in specific medical treatments (49).

### Limitations of the Study

This study was planned as a pilot one: the sample size (*N* = 8) was relatively small, thus reducing the statistical power of the study. Nevertheless, significant differences were observed in the comparison of the FB vs. noFB load data, and as suggested by Friston (50), significant results based on a small sample may indicate a larger FB influence than the equivalent results with a large sample. Future investigations should include a larger number of participants to confirm these preliminary findings. The possibility of recruiting a larger number of participants is potentially useful to identify which disease could mostly benefit from the use of the auditory FB during gait rehabilitation. In addition by analyzing the time elapsed from the occurrence of the CNS lesion and the progression of the ongoing rehabilitation phases, it could be possible to clarify, within a patient-specific rehabilitation project, the optimal time to introduce the use of this system and to favor the load control during gait. This study was a self-controlled case series with a single experimental session. Therefore, a devoted training period and follow-up examination were absent. It may be interesting to include more training sessions with auditory FB in the rehabilitation context, considering the significant improvement observed with a single session, as well as the positive experience reported by both subjects and PhTs. Despite these limitations, our results indicate that sensorized crutches with auditory FB may positively affect participants' adherence to gait objectives.

## Conclusions

These preliminary data suggest that for individuals with CNS lesions, auditory FB significantly improves adherence to instructions to reduce the load on sensorized crutch (es) without affecting gait speed or leading to fatigue. In addition, the participants' experience with FB was positive, and the PhTs' level of satisfaction with the system was substantially high. These positive responses could potentially facilitate collaborations between participants and PhTs.

## Data Availability Statement

The raw data supporting the conclusions of this article will be made available by the authors, without undue reservation.

## Ethics Statement

The studies involving human participants were reviewed and approved by Comitato Etico Indipendente Fondazione Santa Lucia. The patients/participants provided their written informed consent to participate in this study.

## Author Contributions

FT, MLo, NLT, IP, AB, MLa, SP, VS, and MMo contributed to the conception and design of the work. MMa, MLo, and FT contributed to the recruitment of participants. MLa, SP, and MG developed the crutches and the software for the auditory feedback. FT, MLo, NLT, IP, and AB performed experimental sessions. FT, MLo, NLT, FB, AB, and IP worked on data collection, analysis, and interpretation. FT, MLo, NLT, FB, AB, IP, and SP wrote the manuscript and MLa, MG, MMa, VS, and MMo revised it. For this paper the engineering, medical and rehabilitation spheres were combined with the participation of two rehabilitation partners (Fondazione Santa Lucia and Fondazione Turati) and two engineering partners (Campus Bio-Medico University of Rome and University of Brescia). All authors approved the final version of the manuscript.

## Conflict of Interest

The authors declare that the research was conducted in the absence of any commercial or financial relationships that could be construed as a potential conflict of interest.
